# Reactive Oxygen Species Mediated Bacterial Biofilm Inhibition via Zinc Oxide Nanoparticles and Their Statistical Determination

**DOI:** 10.1371/journal.pone.0111289

**Published:** 2014-11-17

**Authors:** Sourabh Dwivedi, Rizwan Wahab, Farheen Khan, Yogendra K. Mishra, Javed Musarrat, Abdulaziz A. Al-Khedhairy

**Affiliations:** 1 Department of Zoology, College of Science, King Saud University, Riyadh, Saudi Arabia; 2 Department of Chemistry, Aligarh Muslim University, Aligarh, India; 3 Functional Nanomaterials, Institute for Materials Science, University of Kiel, Kiel, Germany; 4 Department of Agricultural Microbiology, Faculty of Agricultural Sciences, Aligarh Muslim University, Aligarh, India; University Hospital of the Albert-Ludwigs-University Freiburg, Germany

## Abstract

The formation of bacterial biofilm is a major challenge in clinical applications. The main aim of this study is to describe the synthesis, characterization and biocidal potential of zinc oxide nanoparticles (NPs) against bacterial strain *Pseudomonas aeruginosa*. These nanoparticles were synthesized via soft chemical solution process in a very short time and their structural properties have been investigated in detail by using X-ray diffraction and transmission electron microscopy measurements. In this work, the potential of synthesized ZnO-NPs (∼10–15 nm) has been assessed *in-vitro* inhibition of bacteria and the formation of their biofilms was observed using the tissue culture plate assays. The crystal violet staining on biofilm formation and its optical density revealed the effect on biofilm inhibition. The NPs at a concentration of 100 µg/mL significantly inhibited the growth of bacteria and biofilm formation. The biofilm inhibition by ZnO-NPs was also confirmed via bio-transmission electron microscopy (Bio-TEM). The Bio-TEM analysis of ZnO-NPs treated bacteria confirmed the deformation and damage of cells. The bacterial growth in presence of NPs concluded the bactericidal ability of NPs in a concentration dependent manner. It has been speculated that the antibacterial activity of NPs as a surface coating material, could be a feasible approach for controlling the pathogens. Additionally, the obtained bacterial solution data is also in agreement with the results from statistical analytical methods.

## Introduction

Increased resistance of bacteria against antibiotic medicines is a global health concern. Bacterias are shown to develop resistance to a majority of commercially available antibiotics. Some bacteria produce slime, which is responsible for bacterial adhesion and formation of biofilms on artificial surfaces. Most of the wound infections often including the Gram-positive (+ ve) *Staphylococcus aureus*, *S.epidermidis*, and Gram-negative (-ve) *Pseudomonas aeruginosa* [1]. The pathogen *Pseudomonas aeruginosa* is also known for producing secondary metabolite [2]. These organisms are found to exhibit quorum sensing and produce strong biofilms. The biofilms are surface attached microbial communities embedded in their own microbial-originated matrix of protective and adhesive extracellular polymeric substances (EPSs), mainly polysaccharides, lipids and proteins resistant to antimicrobials [3]. The upcoming approach towards control of biofilms formation involves nanomaterials, which inhibit bacterial adhesion and biofilm formation. NPs with biocidal properties are emerging as new and promising antimicrobial agents as bacteria are less likely to develop resistance against metal NPs than conventional antibiotics. NPs can serve as effective bactericidal materials [4]–[5] and antimicrobial activity of Al_2_O_3_, Fe_2_O_3_, CeO_2_, ZrO_2_ and MgO against pathogenic microorganism (*Pseudomonas sp., Enterobacter sp., Klebsiellasp, morganii* and *S. aureus*) has already been tested by Ravikumar et al., [6]. In another study the effect of silver NPs against water borne pathogens namely *Pseudomonas aeruginosa* and *Vibrio cholerae* was tested [7]. Also, the effect of ZnO, CuO, Ag, Au and Bi on dental caries causing bacteria *S.mutans* has been widely studied [8]–[10]. The inherent property of bactericidal activity of NPs has prompted us to investigate the role of ZnO-NPs as an effective surface coating antimicrobial agent. Among various metal and metal oxide nano- and microstructures, zinc ions (Zn^2+^) of zinc oxide has potential to interact with protein, free ions (Zn^2+^) and can also be an effective target in HSV-1 pathogenesis. The tetrapod like structures of ZnO synthesized by flame transport synthesis process capacity to block the entry and spread of HSV-2 virus into target cells and have ability to neutralize HSV-2 virions [11]–[13]. Towards this direction, several instrumentation and methods have been applied to observe the accuracy and reliability of bacterial strain solution result such as inductively coupled plasma atomic emission spectrometery (ICPAES), photoluminescence (PL) spectroscopy, atomic absorption spectrophotometer (AAS), X-ray fluorescence spectrometery (EDX). The used techniques are more time consuming and less sensitive to determine at low concentrations, very costly, insufficient for selectivity and sensitivity [14]. Over various applied techniques for different purposes, UV-visible spectrophotometric determination are very less time consuming, simple and cost-effective, high reproducibility, sensitivity of quantitative evaluation of colored and colorless solutions with significant economical advantages due to strictly defined standard of quality and quantity at low concentration levels (∼ microgram) mainly depend upon adequate method[15]. In this study, we report the synthesis of ZnO-NPs using soft chemical/solution process. The size and structure of these NPs were determined with the standard characterization techniques such as transmission electron microscopy (TEM), atomic force microscopy (AFM) and X-ray powder diffractometery (XRD). The biocidal activities of NPs have been investigated on total bacterial growth and bio-film formation with aim of elucidating the efficacy of ZnO-NPs, as future nanoantibiotics in biomedical applications. Additionally, there is a growing demand to determine the most appropriate and exact analytical methods for statistical analytical regression analysis to monitor the used nanostructures.

## Materials and Methods

### Experimental

#### Synthesis of Zinc Oxide Nanoparticles (ZnO-NPs)

The ZnO nanoparticles were successfully synthesised using zinc acetate di-hydrate (Zn(Ac)_2_. 2H_2_O, 0.3M) and xylene (C_8_H_10_) in 100 mL of methanol (MeOH) under constant stirring. The chemicals were purchased from Aldrich Chemical Co. Ltd and used without further purification. In a typical experiment: The zinc acetate dihydrate (Zn(Ac)_2_. 2H_2_O) and xylene (C_8_H_10_) were diluted in equal (1∶1) ratio in MeOH and stirred the solution for 30 min to complete dissolution of acetate salt. The pH of this solution was measured and it was reached at ∼6.86. The solution was transferred to the three necked refluxing flask and was refluxed at ∼65°C for 6 h and then air cooled at room temperature. With rise in refluxing temperature, the transparent solution was changed in to white suspension and white precipitate was achieved in 6h. No precipitate was observed before 6 h. The obtained precipitate has been washed several times with MeOH, ethanol and acetone to remove the ionic impurities and dried at room temperature in a glass petridish. The white powder sample was characterized in terms of their morpholo gical, chemical and analytical properties.

#### Characterization of ZnO-NPs

The synthesized ZnO-NPs were characterized in terms of their morphological and crystalline properties with the standard characterization tools such as XRD (Rigaku, Japan) and TEM (Jeol JSM-2010, Japan). The crystallinity of NPs were observed via XRD (powder) with Cu_Kα_ radiation (λ = 1.54178 Å), Bragg angle ranging from 20° to 65° and 6°/min scanning speed. For TEM measurement, white powder of ZnO-NPs was sonicated in an ethanol for 10 min and then a carbon coated copper grid (400 mesh) was dipped into this dispersion solution and dried at room temperature. The dried copper grid was fixed in a sample holder and analyzed at 200 kV in the TEM. The surface topographical observations of the obtained ZnO-NPs were examined using atomic force microscopy (AFM, Veeco Instruments, USA) and the analysis was performed by running the machine in non-contact mode. The characterization of ZnO-NPs was done by observing the patterns appeared on the surface topography and analyzing the AFM data. Tapping mode imaging was implemented in ambient air by oscillating the cantilever assembly at or near the cantilever's resonant frequency using a piezoelectric crystal. The topographical image was obtained in tapping mode at a resonance frequency of 218 kHz. Characterization was done by observing the patterns on the surface topography and data analysis through WSXM software.

### Well Diffusion Assay

The NPs were tested for the antimicrobial activities against strains *Pseudomonas aeruginosa*. In a typical experiment: the pathogenic bacteria were maintained on nutrient agar in petri-dish, and ∼10 mm wells were cut from the agar layer using sterile 1 mL micropipette tip after the solidification of agar layer. The 100 µL of each solution of the prepared ZnO-NPs was poured in every well. The control (without ZnO-NPs) solution was also plated for to check their colony assay forming. Plates were incubated overnight at 37°C and the colonies were counted. After incubation, the diameters of inhibition zone (mm) were measured.

### Biofilm Inhibition Assay

This is a modified method followed by Fletcher et al., [16]. The inoculums used for this assay were maintained and sub-cultured into Nutrient broth (NB) medium along with ZnO-NPs solution (5-100 µg, respectively) to be tested for biofilm inhibition. Culture samples of 1 mL were then aliquoted into 6well plates. The microtiter plates were incubated under stationary conditions at 37°C for 4 days. At an interval of every 28 h for 4 days, the medium was discarded from each well. However, discarding the bacterial culture medium will not disturb the biofilm developed on the walls of tubes, which were analyzed subsequently. The well were then treated with a 0.1% aqueous solution of crystal violet (1 mL) and allowed to stand at ambient temperature for 30 min. The solution was washed with water and the remaining stain was solubilized with 2 mL of 95% ethanol. Biofilm inhibition has been quantified by measuring the OD570 for each tube by transferring the ethanol solution into quartz cuvette for analysis. Without ZnO-NPs solution, inoculums used as control. The assays were performed in triplicate manner.

### Intracellular Measurement of Reactive Oxygen Species (ROS) by ZnO-NPs

ROS was measured with an oxidation-sensitive fluorescent probe 2,7-dichlorofluoresce indiacetate (DCFH-DA)[17]. This DCFH-DA passively diffuses through the cell membrane into the cell and is deacetylated by esterases to form non-fluorescent 2,7-dichlorofluor-escein(DCFH). The DCFH reacts with ROS to form the fluorescent product 2,7-dichlorofluorescein (DCF) [17], which is trapped inside the cell making fluorescent. For this study, *Pseudomonas* was cultured to 10^8^ cfu/mL, and cells were washed three times with fresh medium. DCFH-DA was mixed with the cultures at a ratio of 1∶2000 and the mixture was shaken for 30 min at 37°C. After incubation the bacteria were pelleted by centrifugation and washed two times to remove the DCFH outside the cell. The cleaned cells were exposed to ZnO-NPs at increasing concentration from 10–100 µg/mL. The fluorescence intensity of DCF was measured by fluorescence spectrophotometer at an excitation wavelength of 488 nm and at an emission wavelength of 535 nm. Formore confirmation of bacterial inhibition with NPs, the samples were analyzed with Bio-TEM (Hitachi, Japan) with resolution: 0.2 nm(lattice image) at 100 KV.

### Analytical procedure

For the analytical procedure: ZnO-NPs (1×10^−2^ M), pH 6.86 (1.2 mL), bacterial suspension solution was mixed with distilled water and was prepared in 10 mL of volumetric flask. The absorbance was recorded against the bacterial solution (blank) and the wavelength (nm) was determined by using UV-visible spectrophotometer. The concentration of NPs and pH buffer solution range was optimized at room temperature.

## Results

### Morphological (TEM), crystalline (XRD) and surface topographical (AFM) study of grown ZnO-NPs

**Figure 1:**
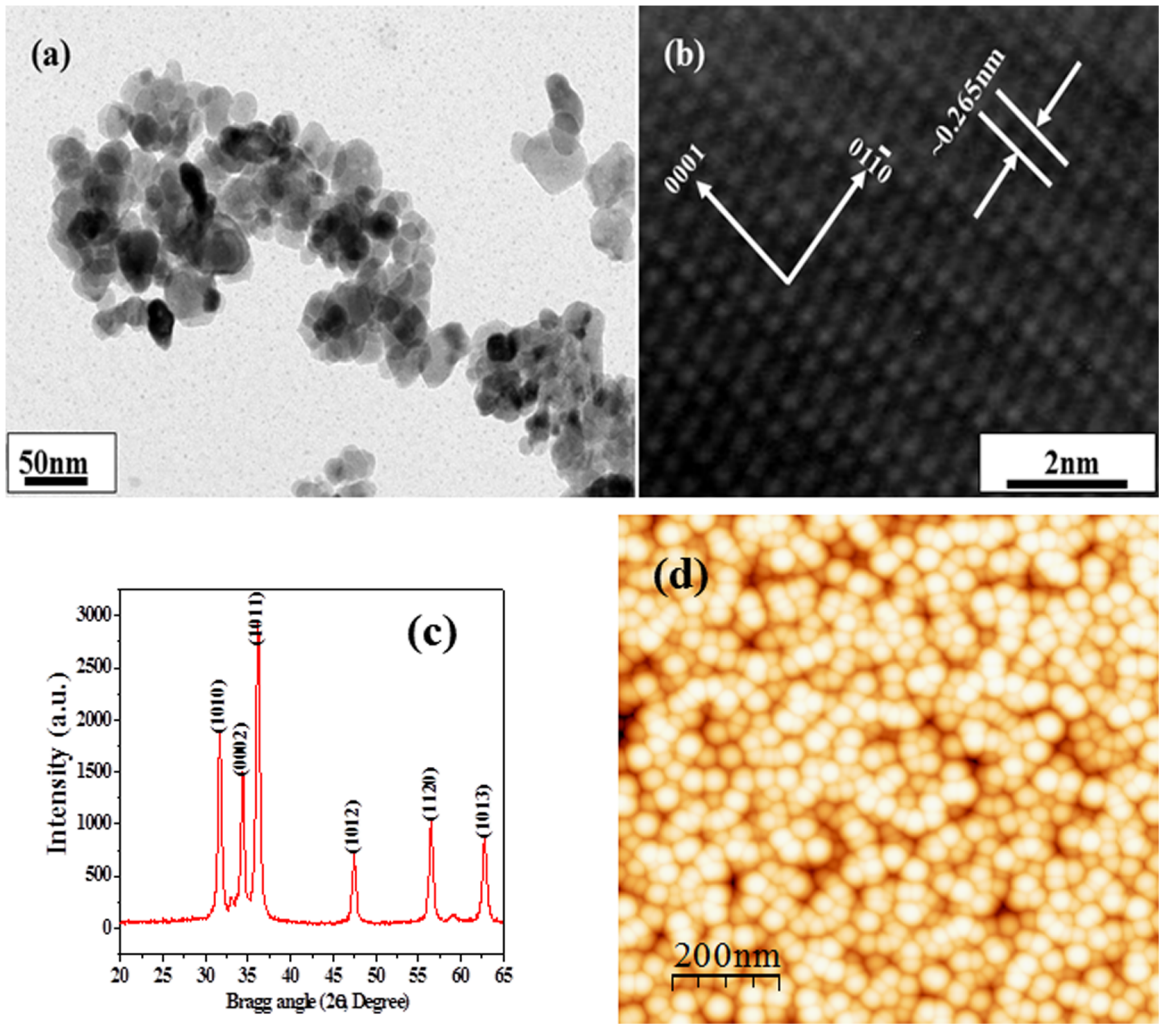
(a) Shows the low magnification TEM image of the ZnO-NPs, (b) HR-TEM image shows the difference between two lattice fringes, which is about 0.265 nm and consistent with The HR-TEM observations, which indicate the crystallinity of the synthesized products. (c) Typical XRD pattern of grown ZnO-NPs. whereas (d) shows the AFM image of ZnO-NPs.

The morphology of the synthesized ZnO-NPs was analyzed with high resolution TEM and corresponding results are shown in Figure 1. From the low magnification TEM image (figure 1a) of ZnO-NPs, it can be clearly seen that each NP particle exhibit smooth surface with spherical shaped morphology. The average size of NPs obtained is in the range of ∼10–15 nm. Figure 1(b) shows a high resolution TEM image of NP. The lattice fringes are separated by ∼0.265 nm which is equal to the lattice constant of wurtzite ZnO phase. The size of the grown nanoparticles was further analyzed with X-ray diffraction pattern (XRD) and it demonstrates the crystallinity of the prepared white powder under defined condition earlier. The XRD spectrum (fig.1c) clearly shows the diffraction peaks in the pattern indexed as ZnO with lattice constants a = 3.249 A and c = 5.206 Å, and it is in good agreement with the available Joint Committee on Powder Diffraction Standards (JCPDS, 36–1451). No other peak related to impurities was observed in the spectrum within the detection limit of the X-ray diffraction, which further confirms that the synthesized powder consists of almost pure ZnO nanoparticles. The intensity of the peaks (<1010>, <0002>, <1011> for the ZnO-NPs) defines the crystalline property of the grown materials. As the intensities of the peaks increases the crystallinity of the material increases. The size (diameter) of the NPs has been calculated with well-known Scherer formula [18] with full width at half maximum (FWHM) of X-ray diffraction pattern. The average value of particle sizes is ∼10–15 nm. The morphology and size of the NPs were further validated by atomic force microscopy analysis. Figure.1d shows the AFM image of ZnO-NPs obtained on scanning probe microscope in tapping mode, under ambient conditions. The average size of each NP and roughness (Ra) of surface were determined to be ∼10 and 15 nm, respectively using the WSXM and SPIP software's.

### Antimicrobial activity and biofilm inhibition by ZnO-NPs

**Figure 2:**
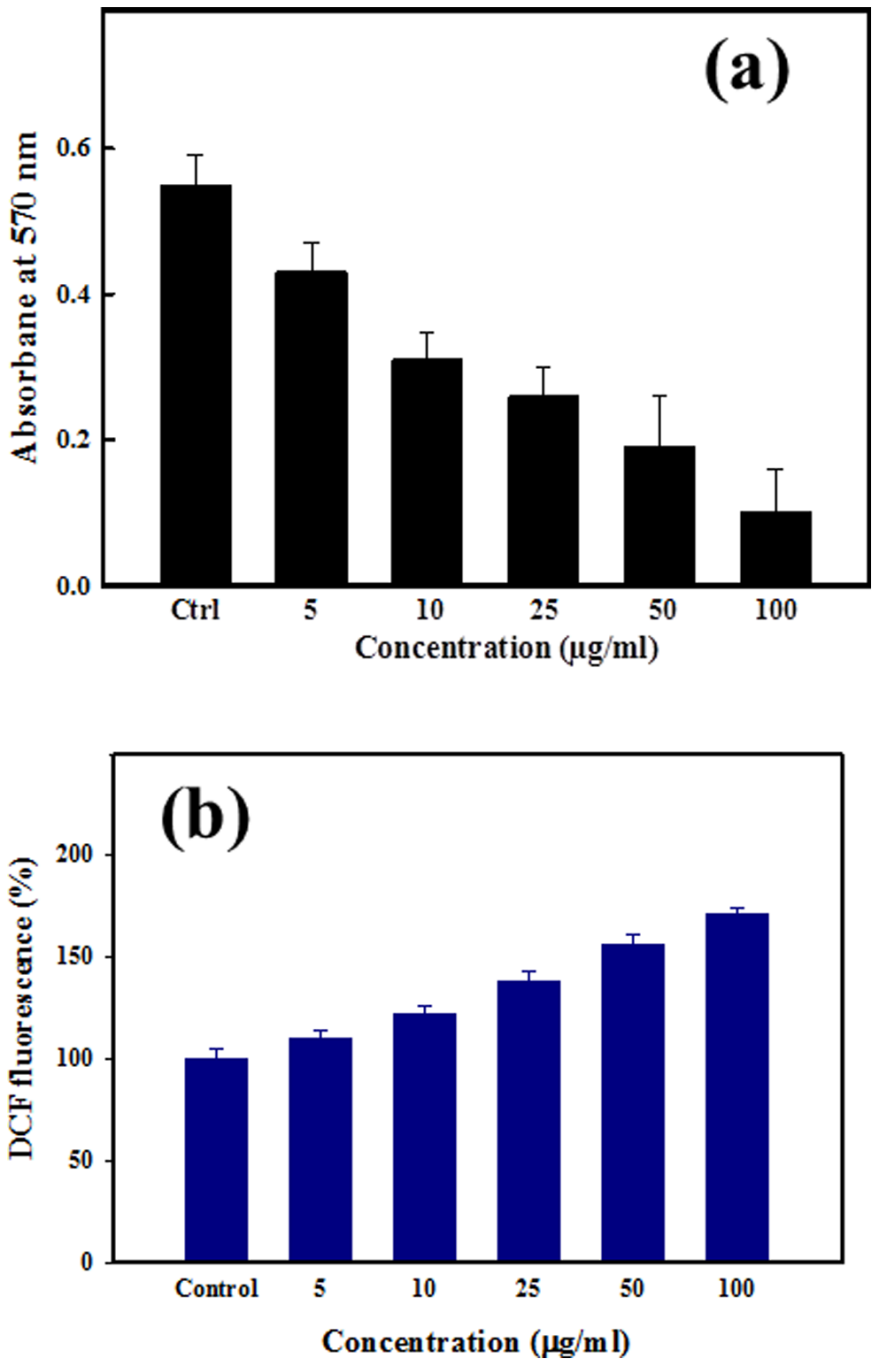
(a) Inhibition on *P.aeruginosa* biofilm formation at different concentration of ZnO-NPs. (b) ZnO-NPs induced oxidant generation in bacterial cells treated with various concentrations of ZnO-NPs (0, 5, 10, 25, 50 and 100 µg/mL) incubated for 24 h.

**Figure 3:**
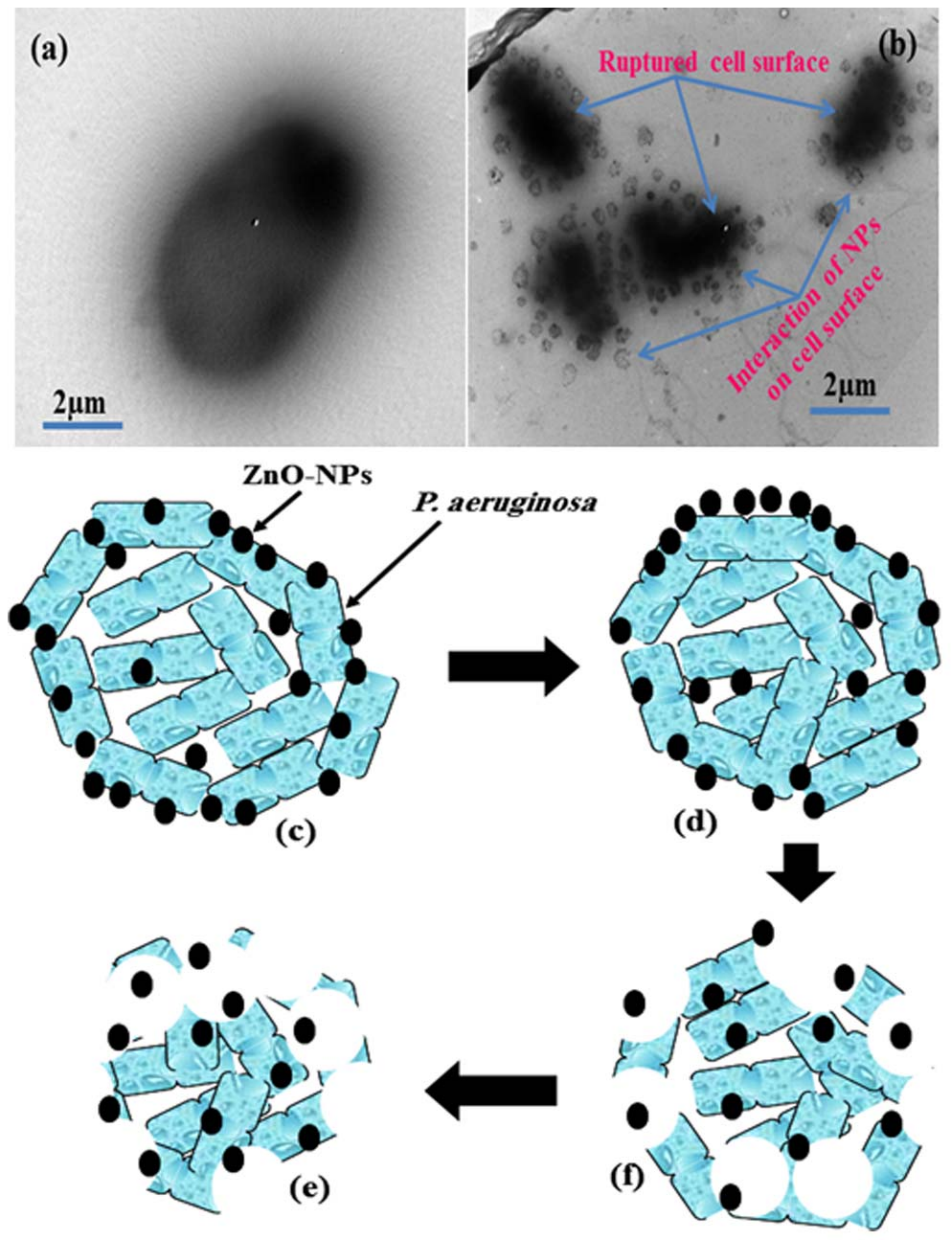
Bio-TEM images of: (a) *P.aeruginosa* (b) *P. aeruginosa* with ZnO-NPs. (c-f) Possible proposed pictorial mechanism.

The obtained data (Table 1) revealed that the bactericidal activities of NPs against tested *P.aeruginosa* via agar diffusion technique. The result shows the good zone inhibition with the use of prepared ZnO-NPs against *P. aeruginosa*, incubated at 37°C overnight and the colonies were counted (Table 1). The results represent that best zone inhibition with NPs against *P.aeruginosa* (16 mm), at 100 µg/mL of ZnO-NPs concentration. For the inhibition of biofilm formation by NPs, crystal violet staining of biofilm was employed to measure the adhesion of biofilm bacteria on microtiterplate. The biofilm formation was significantly inhibited at 50 and 100 µg/mL of ZnO-NPs (figure. 2a). A recent study demonstrated ZnO-induced inhibition of *S.mutans* on oral surface [19]. The major factor for the inhibition of bacteria is reactive oxygen species (ROS), which produced when foreign particles interacts with bacterial solution. We have investigated the ROS in bacteria and the data can be seen in Figure. 2b, the fluorescence intensity increased, indicated an increased ROS level. Thus, it may be inferred that the mechanism of ZnO-NPs antibacterial activity was through increased ROS formation, which indicates the increased antibacterial activity of ZnO-NPs. The mechanism/relation between the bacteria and ZnO-NPs and its anti-bacterial activity have been further elucidated via Bio-transmission electron microscopy (Bio-TEM) images. Figure 3(a–b). Show the TEM images of the tested bacteria *P.aeruginosa*, with ZnO-NPs after 24 h of incubation. In case of *P. aeruginosa*, it is very clear from the images that the NPs have attached at first to the outer membrane of the cell (figure 3(c) and it further entered (figure 3(d) into the cell completely (figure 3(e), which might have lead to cell death (figure 3(f). The pictorial diagrammatic representation of toxicity of ZnO-NPs against biofilm forming bacteria has also been shown in Figure3(c–f).

**Table 1:** Zone of inhibition of Bacterial growth of *P.aeruginosa* with increasing concentration of ZnO-NPs done in triplicate manner.

Concentration (µg/ml) of ZnO-NPs	Zone of inhibition(mm)
Control	Nil
5	6±0.1
10	6±0.5
25	8±0.1
50	12±0.4
100	16±0.4

### Analytical determinations of ZnO-NPs

**Figure 4:**
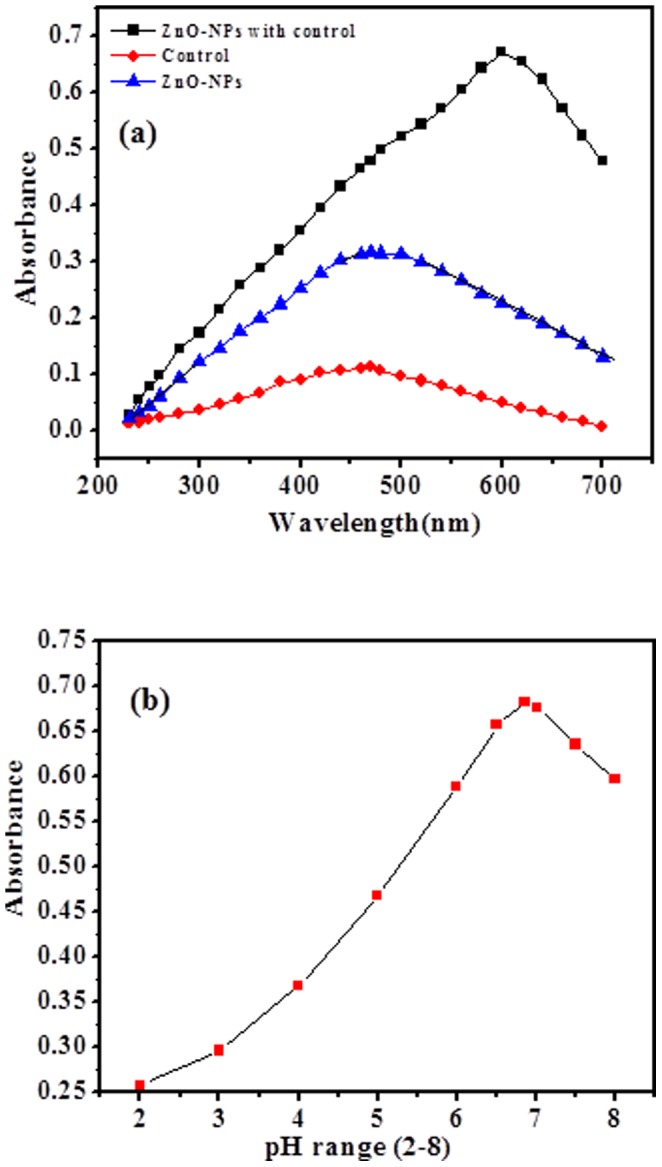
(a) The absorption spectra of nanoparticles of ZnO, control and nanoparticles of ZnO with control (*P.aeruginosa*). (b) Optimizion of pH range (2 to 8.0).

Analytical method applied for quantitative analysis to measure the concentrations of target analytes in biological specimens. The dissolving powder material (ZnO-NPs) calibrated analytical balances made to the resulting concentration for accuracy, precision, limit of detection and quantitations recognized to material purity. The regression analysis of the calibration data were optimized and validated by statistical analytical parameters as per guidelines of International Conference on Harmonisation (ICH) [20]. The valid statistical analytical techniques used in proposed method and obtained results established by UV-visible spectrophotometer which provided quantitative and qualitative satisfactory results. The absorption spectra show ZnO-NPs absorbance was recorded at 600 nm wavelength as can be seen in Figure 4.

The linear calibration graph was constructed ZnO-NPs by plotting absorbance against concentration (conc., 0.5–2.0 µg/mL), which gives linearity and optical regressive data's. The molar absorptivity of ZnO-NPs (3.35×10^2^ L/(mol.cm)) of the resulting colored sample solution indicates sensitivity of the proposed method (Table 2). The minimum concentration of nanomaterials and need to verify works reliability of the proposed methods. The limit of detection and limit of quantitation is a parameter of “limit test” and expected to produce a response, which is significantly different from that of a blank. Therefore, the lowest amount of nanomaterials (ZnO-NPs) in a bulk sample can be detected and compared to know the concentration of nanomaterials against the blank reagent. The limit of detection and limit of quantitation was calculated by following formula as:

**Table 2:** Statistical analytical parameter for the determination ofZnO-NPs.

S.N.	Parameters	ZnO-NPs-bacteria
1.	Color intensity time	1 day
2.	Temperature of solutions	25±1°C
3.	Wavelength(nm)	600
4.	Spectra range (nm)	200–700
5.	pH	0.686
6.	Beer's law limit (µg/mL)	0.5–2.0 µg/mL
7.	Molar absorptivity (L/mol/cm)	3.40×10^2^
8.	Linear regression equation	A = –0.0027+0.3324C
9.	± tsa	6.11×10^−3^
10.	± tsb	4.20×10^−3^
11.	Correlation coefficient (r)	0.9942
12.	Variance(So^2^) of calibration line	3.38×10^−4^
13.	Detection limit (µg/mL)	0.182
14.	Quantitation limit (µg/mL)	0.553

Where ± t_Sa_ and ± t_Sb_ are confidence limits for intercepts and slope respectively.


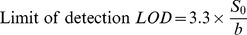



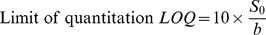


Where (S_0_)  =  Standard deviation of the blank, (b) Residual standard deviation of the calibration line, or standard deviation of the intercept.

The obtained calibration curve of slope (b), and standard deviation of the intercept (S_0_) analyzed at the time to validate level, acceptable accuracy and precision under the quantified operational conditions of proposed method. The limit of quantitation is around of triple the limit of detection. The analytical method was applied to verify the proposed methods works reliability. Typical validation parameters are used for accurate concentration of analytes (ZnO-NPs) or sample solution and calculate with the help of statistical parameters andtheir empirical formula provided more constantly values to ensure that they are fit for proposed method. Table 2. Indicates recovery and relative standard deviation (RSD) experiments results which is the evident that recoveries accurate and satisfactory[20]–[22].

### Optimization and Validation

**Figure 5:**
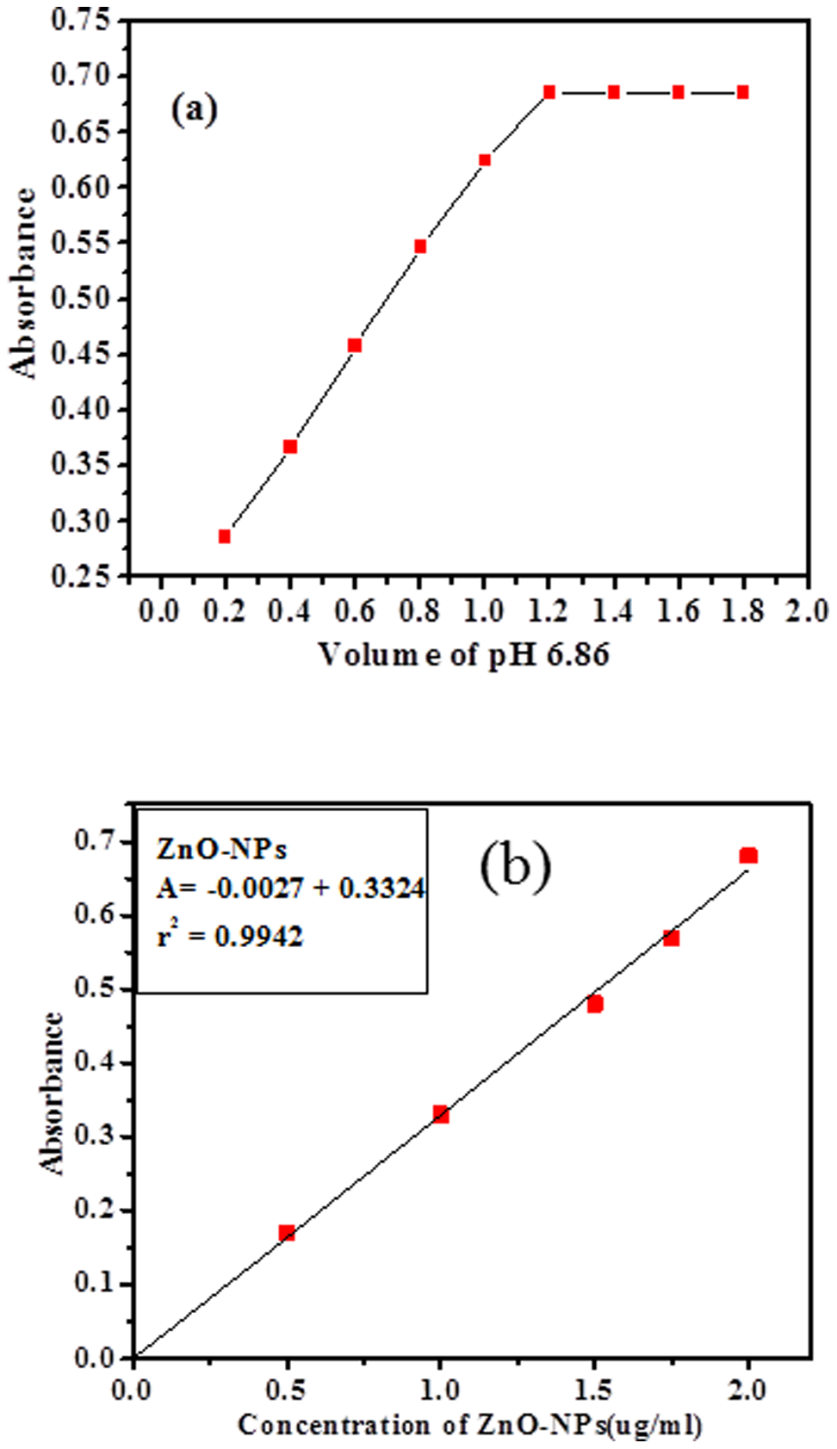
(a) pH (6.86) volume graph, (b) Linear calibration graph of nanoparticles (NPs) with (*P.aeruginosa*) bacteria.

As per guidelines of International Conference on Harmonisation (ICH) [20], optimization and validation are the important parameters, which applied todetermine minute concentration level of analytes (ZnO-NPs) present in sample solutions. The minute concentration level is major requirements of biological samples and provided quality control of bacteria growth. The pH buffer solution range, pH volume (Figure.4b and 5a) and concentration of ZnO-NPs optimized at low temperature, which shows high effect on growth of bacteria (*P.aeruginosa*). The ability of analytical procedure (within a given concentration range) to get test results, which are directly proportional to the concentration of analyte (ZnO-NPs) in the sample solutions. Beer's law was obeyed (conc., 0.5–2.0 µg/mL) as can be seen in Figure.5 b. The optical regressive characteristics results such as: values of correlation coefficient (ZnO-NPs  = 0.9942), apparent molar absorptivity (ZnO-NPs  = 3.40×10^2^ L/(mol.cm)), detection limits (LOD) (NPs  = 0.182 µg/mL) and quantitation limit (LOQ)(NPs  = 0.553 µg/mL), respectively as can be seen in (Table 2). The multiple day's analysis (intra-day and inter-day precisions) at three concentration levels (0.489, 1.45 and 2.0 µg/mL)wascarried out within the same day and five consecutive days. The intra-day and inter-day RSD (%) values ranged (ZnO-NPs  = 0.92–1.54% and 0.66–1.02%) respectively. The results are summarized in Table 3. The recoveries (%) of ZnO-NPs (99.17–100.41%) represented good accuracy of the proposed analytical method [20]–[22].

**Table 3:** Test of precision of the proposed method for ZnO-NPs.

Parameters	Intraday			Interday		
Concentration taken (µg/mL)	0.489	1.45	2.0	0.489	1.45	2.0
Concentration found (µg/mL)	0.490	1.45	2.0	0.491	1.43	1.99
Standard deviation (µg/mL)	0.006	0.022	0.018	0.003	0.001	0.014
Recovery (%)	100.20	100.41	100.00	99.59	99.17	99.50
Relative Standard deviation	1.34	1.54	0.92	0.66	1.02	0.72

## Discussion

**Figure 6:**
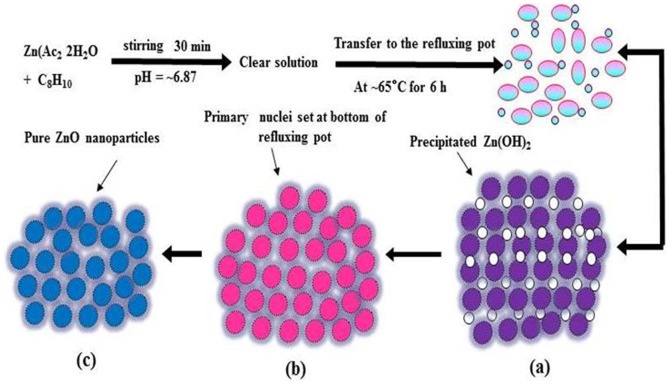
Possible schematic presentation for the formation of ZnO-NPs.

The fabrication of nanoscale (<100 nm) NPs and their applications in industrial and biomedical area are increasing rapidly due to their high surface area and larger catalytic property [23]-[24]. Additionally, their biocompatible nature makes valuable materials for the broad biomedical applications such as drug delivery, cytotoxicity, cell, DNA and as an antibiotic against bacterial population damage etc [25]. The nanoparticles of ZnO have been prepared with soft chemicalsolutionprocess using xyleneand characterized well with the tools such as X-ray diffraction, TEM equipped with the HRTEM and atomic force microscopy (AFM), which are used to identify the formation and topography of grown NPs. The crystalline nature with wurtzite phase of ZnO-NPs was confirmed with X-ray diffraction pattern. The transmission electron microscopy (TEM) showed that NPs were nearly spherical in shape, smooth surface and with an average spherical diameter of 10 to 15 nm in an aggregated form. The high resolution TEM also confirmed that the grown NPs are crystalline and having lattice spacing (∼0.265 nm) equal to pure wurtzite phase ZnO [26]. The surface topography has also been checked via atomic force microscopy (AFM) and result are clearly consistent with the TEM observation. The average diameter of each NP are in the range of 10-15 nm and spherical in shape. The obtained NPs are an optically active materials and having similar band gap to the commercial zinc oxide powder (3.37eV), which is a characteristic band of wurtzite hexagonal pure ZnO. On the basis of characterizations, we have also presented the brief proposed mechanism for the formation of spherical shaped ZnO-NPs. As the, solution of xylene was mixed to the source material of NPs (zinc acetate di-hydrate) under continuous stirring with solvent methanol (MeOH). The solution appeared clear without precipitate at pH ∼6.86 and it transferred to the refluxing pot and refluxed the solution at 65°C. When the refluxing time was increased, a white colored precipitate started to form and the formation process was completed in 6 h. Here, we may assume that the small nuclei of NPs formed in the refluxing pot due to reaction of zinc acetate and alkyl group of xylene. In this reaction, solvent MeOH directly reacts and it forms Zn(OH)_2_. The hydroxide of zinc ions (Zn^2+^) exist in the solution as an ionic form and at higher refluxing temperature zinc ions and hydroxyl (OH^-^) ions changes in to pure ZnO and water molecule. The reaction impurities/by product from the reactions were removed by washing with alcohol and room temperature drying process. It is assumed that initially formed Zn(OH)_2_ in the solution, nuclei gets set to the bottom of refluxing pot and after acquiring sufficient thermal energy from the refluxing pot it forms small active molecules of ZnO (figure6a-c) [27]–[29]. The obtained spherical shaped NPs was tested for the antimicrobial activities against strains *Pseudomonas aeruginosa* via Well diffusion study. Our measurement shows the best zone inhibition withNPs against *P.aeruginosa* (16 mm), at 100 µg/mL of ZnO-NPs concentration. To know the adhesion of biofilm crystal violet staining was employed on bacteria on microtiterplate. The biofilm formation was significantly inhibited at 50 and 100 µg/ml of ZnO-NPs. Our results also exhibited inhibition of biofilm formation at 100 µg/ml of ZnO. Control cultures exhibited a gradual increase in the absorbance due to crystal violet concentration, which is directly proportional to the biofilm formation. The data revealed greater toxicity of NPsin concentration dependent manner with cell wall disruption and higher membrane permeability. These results are also corroborate with our previous investigated by Wahab et al [30]–[32]. It has also been reported that metal nanoparticles induced a significant rise in reactive oxygen species (ROS) in cell lines that elicited toxic effects related to oxidative stress [17], [32]. ROS were possibly produced when respiratory enzymes were inhibited through the interaction of metal ions with the thiol group of the enzymes [33]. An earlier study reported that ROS in bacteria primarily resulted from the autoxidation of NADH dehydrogenase II in the respiratory chain [34]. To understand the mechanism of antibacterial activity, ROS generated in the presence of ZnO-NPsunder different temperatures were monitored with oxidation-sensitive fluorescent probe DCFH-DA that passively diffuses through the cell membrane into the cell. The ZnO-NPs affect the cell membrane and leads to ROS formation, DCFH-DA inside the cell reacts with ROS and converted into fluorescent by-product DCF. The ROS level was analyzed in the original bacteria in comparison with ZnO-NPs-exposed bacteria (F–F0) using cell fluorescence intensity. We speculate that the action of ZnO-NPs is broadly similar to that of zinc ion. It may be anticipated that a bacterial cell in contact with ZnO-NPs takes in zinc oxide ions, which inhibit respiratory enzyme(s), facilitating the generation of ROS and consequently damaging the cell. ZnO-NPs and their ions (Zn^2+^) can produce free radicals, resulting in induction of oxidative stress (i.e., reactive oxygen species; ROS). The produced ROS can irreversibly damage bacteria (e.g., their membrane, DNA, and mitochondria), resulting in bacterial death.

The present work also providesthe validation of analytical methods perform to test for the bacteria growth inhibitors at a very low concentration and statistical analytical parameters employed for zinc oxide nanostructures such as Mean (X) was measured from five independent determinations for all data points. Standard deviation (SD), relative standard deviation (RSD) and confidence limit (C.L.) at 95% were calculated in order to verify the validity of experimental data's. The nanoparticles absorb UV-light andexhibit maximum absorbance at wavelength ∼600 nm. The used concentration for grown nanoparticles, which gives linearity and regressive data's. The maximum wavelengths of absorption spectra depend on the nanostructures materials, particles size and shapesand show molar absorptivity. The obtained NPs structures are suitable for inhibition of bacteria growth at minute concentration levels [35]–[36]. The performance of the proposed method is free from different type of errors such as sampling error, dilution error, plating error, incubation error, and operator error. The proposed method used analytical for the technique to determine or authenticate a number of precise routine characteristics properties such as sensitivity, specificity, accuracy, precision, trueness, reproducibilityand ruggedness to ensure that the results are fit for the inhibition of bacterial growth. The optimized concentration of ZnO-NPs is highly affected on the bacteria growth and standard analytical techniques define the quality of the results. The satisfactory data's are obtained from UV-visible spectroscopy, provides qualitative and quantitative results. The analytical parameters are authenticated under studiesof ICH for validation and organization for standardization of analytical procedures [35]–[36].

## Conclusion

The summary of this study concluded that the ZnO-NPs exhibited significant inhibitory activity on bacteria and biofilm formation. The results suggest that the NPs act as the potential antimicrobial agents, and elucidated their effective control on biofilm formation. Further studies are required to understand the mechanism by which the ZnO-NPs inhibit the growth of total bacteria and biofilm formation. Nevertheless, the results support the hypothesis that the ZnO-NPs could serve as an alternative antimicrobial agents. The concentration of ZnO-NPs with are highly affected the growth of bacteria with respect to dose dependent manner. The cell wall of bacteria destroys and completely damaged at low concentration of ZnO-NPs. Generally, accurate measurements of constituents in biological samples are very critical therefore, applied analytical technique and statistical analytical parameters for knowing accurate amount of ingredients fixed by optimization and validation process. The obtained statistically analyzing data's provided good accuracy and precisionwithout interfering of different types of errors such as sampling, dilution, plating, incubation and operator errors. The observations indicates that the analytical technique is authentic technique and decent way to measured data analyzed, provides simplicity, sensitivity, rapidity, purity and quality control growth of bacteria with satisfactory results.
